# Prenatal Alcohol Exposure in Rodents As a Promising Model for the Study of ADHD Molecular Basis

**DOI:** 10.3389/fnins.2016.00565

**Published:** 2016-12-15

**Authors:** Argelia E. Rojas-Mayorquín, Edgar Padilla-Velarde, Daniel Ortuño-Sahagún

**Affiliations:** ^1^Departamento de Ciencias Ambientales, Centro Universitario de Ciencias Biológicas y Agropecuarias, Instituto de Neurociencias, Universidad de GuadalajaraGuadalajara, Mexico; ^2^Departamento de Biología Molecular y Genómica, Instituto de Investigación en Ciencias Biomédicas, Centro Universitario de Ciencias de la Salud, Universidad de GuadalajaraGuadalajara, Mexico

**Keywords:** alcohol, prenatal alcohol exposure, ADHD, fetal alcohol spectrum disorders, animal model

## Abstract

A physiological parallelism, or even a causal effect relationship, can be deducted from the analysis of the main characteristics of the “Alcohol Related Neurodevelopmental Disorders” (ARND), derived from prenatal alcohol exposure (PAE), and the behavioral performance in the Attention-deficit/hyperactivity disorder (ADHD). These two clinically distinct disease entities, exhibits many common features. They affect neurological shared pathways, and also related neurotransmitter systems. We briefly review here these parallelisms, with their common and uncommon characteristics, and with an emphasis in the subjacent molecular mechanisms of the behavioral manifestations, that lead us to propose that PAE in rats can be considered as a suitable model for the study of ADHD.

## Introduction

Prenatal Alcohol Exposure (PAE), causes a pleiad of neurological alterations, that consist in physiological, cognitive, and behavioral abnormalities, which together are identified as “Fetal Alcohol Spectrum Disorders” (FASD) (Sokol et al., 2003; Bertrand et al., 2005; Hoyme et al., 2005), that includes a range of categories, referred by the Institute of Medicine (IOM) (Stratton et al., 1996) as “Alcohol Related Neurodevelopmental Disorders” (ARND); “Alcohol Related Birth Defects” (ARBD); the partial “Fetal Alcohol Syndrome” (pFAS); and finally, the “Fetal Alcohol Syndrome” (FAS), which is the most severe form and includes growth deficiencies and facial abnormalities caused because of alcohol consumption during pregnancy. The severity of the effects that alcohol ingestion produce over embryonic (fetal) development, depends on the stage and the amount of the alcohol, in the form of ethanol (EtOH), that are ingested.

Results from human studies and animal models clearly show that PAE-related abnormalities in physiological function include alterations in the activity and regulation of hypothalamic–pituitary–adrenal (HPA) and hypothalamic–pituitary–gonadal (HPG) axis, as well as modifications in the interactions between these systems (Handa et al., 1985; Lee and Rivier, 1996; Weinberg et al., 2008; Comeau et al., 2015). Likewise, PAE animals display cognitive and behavioral deficits, including delays in learning and memory, and altered responsivity to stressors (reviewed in Hellemans et al., 2010). More specifically, PAE affects different stages of brain development from neurogenesis to myelination, through a variety of mechanisms, including disrupted cell-cell interactions, altered gene expression, oxidative stress, and growth factor signaling disruptions (Reynolds et al., 2011; Riley et al., 2011), that occurs even without severe physical teratogenicity (Riley et al., 2011; Schneider et al., 2011).

In fact, the profound effects that PAE produce on the developing brain gene expression and physiology, results in the cognitive and behavioral effects that ensue. Some recent studies on fetal and neonatal brains have uncovered EtOH-induced alterations in the expression of genes related to energy metabolism, cell adhesion, cytoskeletal remodeling, cell cycle, proliferation, differentiation, apoptosis, as well as neuronal growth and survival, and also nervous system development, free radical scavenging and small molecules metabolism (Hard et al., 2005; Zhou et al., 2011; Kleiber et al., 2012). Together, these results suggest a complex residual “footprint” of neurodevelopmental EtOH exposure that would be useful for identifying mechanisms that underlie the life-long persistence of FASD-related cognitive and behavioral alterations, including potential molecular targets for its treatment (Kleiber et al., 2012).

In children, the PAE has been linked with the key symptoms of the Attention-deficit/hyperactivity disorder (ADHD) (Knopik et al., 2005; Bhatara et al., 2006; Kodituwakku, 2007), which is a brain disorder marked by an ongoing pattern of inattention and/or hyperactivity-impulsivity that interferes with functioning or development (NIH definition)^1^ In animal and human research, there is emerging clinical, neuropsychological, and neurochemical evidence of a link between FASD and ADHD (O'Malley and Nanson, 2002; Kodituwakku, 2007). In this regard, it is important to clarify that although the FASD and ADHD constitute two clinically distinct disease entities, the fact is not negligible that ADHD is the most commonly reported mental health diagnosis in individuals with PAE (i.e., up to 97% of 39 children according to Fryer et al., 2007), and a high percentage of children diagnosed with ADHD have a history of PAE (i.e., up to 41% of 2231 children according to Bhatara et al., 2006). Therefore, and based on a large body of evidence, FASD, caused by PAE, appears to be the leading cause of ADHD (Burd, 2016). Then, there may be common etiological pathways to ADHD and the behavioral phenotype of FASD, alternatively, acquired ADHD secondary to PAE may be due to the effect of alcohol on the developing dopamine (DA) transmitter system (reviewed in Peadon and Elliott, 2010).

There may be multiple pathways to the coexistence of ADHD symptoms and FASD, so there may be different subsets of ADHD and FASD (Oesterheld and Wilson, 1997; O'Malley and Nanson, 2002). Some studies support the idea that ADHD within FASD is a particular clinical subtype with earlier onset, a different clinical and neuropsychologic profile, and a different response to psychostimulant medications. In this review, we will contrast and discuss the more recent evidence that points out a parallelism between ADHD and FASD, as a consequence of PAE, and also highlight the specific differences. Furthermore, we will focus on cellular and molecular aspects subjacent to both pathologies to support that specific protocols of PAE would be useful as a model for the study of some aspects of ADHD.

## Involvement of the hypothalamic-pituitary-adrenal axis in PAE and ADHD

One of the most characterized effects of PAE are the dysregulation of the offspring hypothalamic-pituitary-adrenal (HPA) axis (Lee and Rivier, 1994; Ogilvie and Rivier, 1997; reviewed in Weinberg et al., 2008), increasing sensitivity to stressors and vulnerability to stress-related disorders (Lee et al., 2000). It is noteworthy that also an involvement of HPA axis has been clinically described in groups of children with ADHD (Kaneko et al., 1993; Hastings et al., 2009), as well as in its experimental animal's models, as mouse mutant coloboma (Raber et al., 1997); and WKHA rats (Hendley, 2000). Furthermore, an impaired response to stress has been suggested as a marker to the more developmentally persistent form of ADHD (King et al., 1998; Snoek et al., 2004; Pesonen et al., 2011; reviewed in Johnson, 2015). Finally, genetic evidence has been recently presented for the association of HPA axis and ADHD (Fortier et al., 2013). Taken together, all these evidences point out to a physiological relationship, or at least an important physiological parallelism, between PAE and ADHD.

## Key neurotransmitters systems are affected in PAE and ADHD

Several neurotransmitter systems can be affected by PAE, but the degree of affectation depends on the doses of alcohol, the embryo developmental stage at consumption, and the genetic background, of the parents and the embryo. Also, the damage would vary in different cerebral region. Suggesting that alterations of selective neurotransmitters may be the cause of abnormalities in brain function and behavior found in FASD.

One of the mains affected system by PAE is the cholinergic system, preferentially in the hippocampus. Rats exposed to prenatal alcohol exhibit differential response to cholinergic agonist (pilocarpine and nicotine) or antagonist (scopolamine and mecamylamine) in a delay-dependent memory task, suggesting that alterations in the rat cholinergic system may underlie some of the cognitive deficits observed with PAE (Nagahara and Handa, 1999). Also, it has been demonstrated that perinatal choline supplementation may attenuate alcohol-related behavioral changes by influencing cholinergic systems (Monk et al., 2012). For ADHD, there are evidences that point to a role of the cholinergic system in ADHD cognitive dysfunction (Potter et al., 2006; Sarter and Paolone, 2011) and that emphasize the relevance of the therapeutic potential of nicotinic cholinergic agents for their treatment (Potter et al., 2014). Therefore, it is necessary to develop more enhanced understanding of the nicotinic cholinergic system and its role in ADHD (Childress and Sallee, 2014).

Other highly disturbed is the glutamatergic system, mainly the regionally specific glutamate receptor expression (Bird et al., 2015). Particularly, NMDA receptor subunits are affected. A reduction of approximately 30–50% of NR1, NR2A, and NR2B subunits persists at postnatal day 90 in the barrel field cortex of animals under PAE (Rema and Ebner, 1999). Also, a functional NMDA-NR1 receptors are necessary for the neurotoxic and teratogenic effects of PAE (Deng and Elberger, 2003). Additionally, synaptic NR2B-containing NMDA receptor concentrations are reduced in PAE animals (Samudio-Ruiz et al., 2010), specifically in the agranular insular cortex (Bird et al., 2015). Taken together, these evidences could help to explain EtOH-related alterations in learning and behaviors that depend on cerebral cortex. Regarding AHDH, increases in glutamatergic metabolites were found in the Anterior Cingulate cortex (ACC) and other regions in children with ADHD (Spencer et al., 2004); in addition, dysregulation of the NMDA type of glutamate receptors has been recently reported as being involved in ADHD (Chang et al., 2014). However, a relatively small number of studies have examined the role of glutamatergic dysregulation in pediatric psychiatric disorders, emphasizing the need to increase research in these areas.

Additionally, the DopAminergic (DAergic) system is also affected both in PAE (Druse et al., 1990; Diaz et al., 2014; Naseer et al., 2014) and in ADHD (Curatolo et al., 2010). Reduction of DAergic transmission is considered one of the causal mechanisms of ADHD (Ernst et al., 1998). Furthermore, DAT dysregulation has been directly involved in the pathophysiology of ADHD (Madras et al., 2005; Paloyelis et al., 2010). Correspondingly, one of the main therapies employed for ADHD treatment uses dexamphetamine or methylphenidate, both stimulators of the DAergic system (Spencer et al., 2004). In addition, animal models for the study of ADHD exhibit DAergic system alterations associated with hyperactivity, inattention, and impulsivity (Van der Kooij and Glennon, 2007; Genro et al., 2010). On the other hand, DAergic systems, which have neurocircuitries that overlap the HPA axis, are altered by PAE (Uban et al., 2013). PAE negatively affects DA biosynthesis and transport in midbrain neurons (Szot et al., 1999) and direct regulates DAT function by altering endosomal recycling of the transporter (Methner and Mayfield, 2010); also, PAE alters postnatal development of the spontaneous electrical activity of dopamine neurons in the ventral tegmental area (Choong and Shen, 2004). Taken together, all of these evidences lead us to suggest a physiological parallelism, at least in these three neurotransmitter systems (cholinergic, glutamatergic, and DAergic) between PAE and ADHD.

Finally, other systems are also affected by PAE. PAE reduces the concentrations of some catecholamines, indolamine, and amino acid neurotransmitters in E13 fetal brains (Sari et al., 2010). In also affects the GABAergic system (Volgin, 2008; Zhou et al., 2010; Wang et al., 2013), serotonin (Sliwowska et al., 2014), and opioid receptors (Nizhnikov et al., 2014) in a number of brain regions (Bird et al., 2015 and references therein). Thus, it would be of interest to study these systems under the ADHD condition.

## PAE affects epigenetical modulation of gene expression inducing ADHD phenotypes

The preconception of paternal exposure to EtOH produced ADHD-like behavioral phenotypes in the progeny, such as hyperactivity, inattention, and impulsivity, mediated by increased methylation in the DAT promoter region (Kim et al., 2014). Additionally, it reduced NGH and BDNF in some brain regions and increased EtOH-elicited preference in male offspring (Ceccanti et al., 2016). On the other hand, maternal consumption of EtOH during pregnancy has been classically associated with ADHD (Mick et al., 2002; Knopik et al., 2006) and also affects the epigenotype and phenotype of offspring in mouse (Kaminen-Ahola et al., 2010). The range of clinical phenotypes varies in severity and outcome depending on the level, pattern, and timing of maternal alcohol consumption (British Medical Association, 2007). This affectation is mainly metabolic, by the consumption of the alcohol, but might also involve epigenetical alterations (reviewed in Shukla et al., 2008; Haycock, 2009). There are several factors that must be considered, such as maternal age, amount and frequency of ingested alcohol, stage of use (before, during, or after pregnancy, and even during lactation). Thus, there remains the need for much research these respects.

Thus, it is reasonable to propose that, in the case of alterations caused in the progeny by PAE, a synergic effect can be generated when both parents consume alcohol, prior to conception in the case of the father, and before or during pregnancy and/or lactation in the case of the mother, and that at least part of these effects are epigenetically mediated. Recently, some clues have begun to arise regarding this epigenetical modulation, which results in long-term regulatory change and which induce developmental and behavioral defects that may persist throughout the lifetime of an individual (Table 1). It is still unknown how these changes occur and are maintained, what specifically the consequences of those changes are, and which of these consequences are related with FASD from PAE and ADHD; therefore, epigenetic alterations constitute a new whole avenue of research.

**Table 1:** **Main features shared that suggest a parallelism between ADHD (Attention deficit/hyperactivity disorder) and FASD (Fetal alcohol spectrum disorders)**.

	**FASD (PAE)**	**ADHD**
**NEUROLOGICAL ALTERATIONS**		
Cognitive and behavioral deficits	Delay in learning and memory (Hellemans et al., 2010)	Deficits in learning, cognition (attention), and behavior (hyperactivity/Fmpulsivity) (Casey et al., 2007; Albrecht et al., 2015)
Ongoing pattern of inattention and/or hyperactivity impulsivity	Children with FASD often present with attentional problems similar to those observed with ADHD (Coles et al., 1997; Mick et al., 2002; Jacobson et al., 2011)	
Prospective memory	PAE was related to ADHD, but ADHD was not related to prospective memory performance (Lewis et al., 2016)	
Physiological abnormalities	Affects different stages of brain development from neurogenesis to myelination (Riley et al., 2011; Reynolds et al., 2011), which leads into behavioral and cognitive deficits during youth and adulthood	Children show a core deficit in behavioral inhibition, leading to impairments in working memory, self-regulation, internalization of speech and reconstitution (Barkley, 1997)
**HYPOTHALAMIC-PITUITARY-ADRENAL AXIS (HPA)**		
Disfunction of HPA axis	Alterations in the activity and regulation of HPA and HPG axis (Handa et al., 1985; Lee and Rivier, 1996; Weinberg et al., 2008; Comeau et al., 2015)	Involvement of HPA axis has been clinically described in children with ADHD (Kaneko et al., 1993; Hastings et al., 2009)
Altered responsivity to stressors	Increasing sensitivity to stressors and vulnerability to stress-related disorders (Lee et al., 2000; reviewed in Hellemans et al., 2010)	An impaired response to stress has been suggested as a marker to the more developmentally persistent form of ADHD (King et al., 1998; Snoek et al., 2004; Pesonen et al., 2011; reviewed in Johnson, 2015)
**KEY NEUROTRANSMITTER SYSTEMS AFFECTED**		
Dopaminergic system	Prefrontal cortex is particularly affected by PAE, mainly dopamine system (Juh et al., 2005; Smith et al., 2012; Kim et al., 2013; Uban et al., 2015)	Daergic system is affected (Curatolo et al., 2010). Prefrontal cortex is the region most affected (in regulating behavior and attention) via dopamine transmission (Arnsten, 2007)
DA transporter system (DAT)	PAE direct regulates DAT function by altering endosomal recycling of the transporter (Methner and Mayfield, 2010); PAE decreases DAT binding sites in brain (Druse et al., 1990; Szot et al., 1999): in contrast, in adult rats, ethanol increases the number of DAT binding sites in brain (Jiao et al., 2006)	DAT is involved in ADHD and/or its treatment (reviewed in Mazei-Robinson and Blakely, 2006); High striatal DAT availability in most adults with ADHD (reviewed in Krause et al., 2006)
DA receptors	PAE differentially affects regional expression of DA receptor subtypes (Flores et al., 2011). PAE males exhibited increased dopamine receptor expression in medial prefrontal cortex (Uban et al., 2015)	Polymorphisms of D4 and D5 receptors show a predisposition to develop ADHD (Kustanovich et al., 2004)
Cholinergic system	PAE may disrupt learning and memory in adolescence via a cholinergic mechanism (Perkins et al., 2015); Perinatal choline supplementation may attenuate alcohol-related behavioral changes (Monk et al., 2012)	Involved in ADHD cognitive dysfunction (Potter et al., 2006; Sarter and Paolone, 2011)
Glutamatergic system	PAE reduces NMDA receptor subunits expression (Rema and Ebner, 1999; Samudio-Ruiz et al., 2010; Bird et al., 2015)	Dysregulation of the NMDA receptors has been involved in ADHD (Chang et al., 2014)
Gabaergic system	PAE attenuates Gabaergic inhibition in amigdala, leading to hyperexcitability and anxiety (Zhou et al., 2010; Baculis et al., 2015); reduces Gabaergic neurons in vermis (Nirgudkar et al., 2016) and in cortex (Smiley et al., 2015); and affects cortical Gabaergic neuron migration (Skorput and Yeh, 2016)	Disturbed Gabaergic transmission in hippocampus (Sterley et al., 2016) and in prefrontal cortex (Tzanoulinou et al., 2016) have been involved in ADHD neuropathophysiology. GABAergic inhibitory neurons play a role in the neurobiology of ADHD (Nagamitsu et al., 2015). GAD1 polymorphysm is associated with ADHD (Bruxel et al., 2016)
**CELLULAR FUNCTION**		
Altered gene expression	Down-regulation of 25 genes involved in cell proliferation, differentiation, and apoptosis, none were up-regulated (Hard et al., 2005); down-regulation of 104 genes involved in protein synthesis, mRNA splicing, and chromatin organization, none were up-regulated (Rogic et al., 2016)	Genome wide analysis in human ADHD individuals confirms the complexity and heterogeneity of ADHD etiology (Zayats et al., 2015). Down-regulation of 54 genes and up-regulation of 52 genes related with transcription, synaptic transmission, neurological system process and immune response in animal model of ADHD (De la Peña et al., 2015)
Oxidative stress (OS)	Altered gene expression in OS pathways in the adult hippocampus suggests a novel involvement of OS mechanisms in FASD (Chater-Diehl et al., 2016); PAE dysregulates OS in rats (Brocardo et al., 2012), and in Drosophila (Logan-Garbisch et al., 2014)	OS is increased in children with ADHD (Sezen et al., 2016); ADHD patients present an insufficient response to OS leading to damage (Joseph et al., 2015); Interventions with antioxidant represent potential options for the treatment of ADHD (Lopresti, 2015)
Growth factor signaling disruption	PAE in mice alters NGF and BDNF in brain (Ceccanti et al., 2016)	Higher levels of serum NGF in drug-naive ADHD patients (Guney et al., 2014); disrupting of BDNF signals found in children with ADHD (Liu et al., 2015)
**EPIGENETICAL MODULATION OF GENE EXPRESSION**		
DNA methylation	Alterated DNA methylation program during neurulation (Zhou et al., 2011; and the genomic methylation profiles (Liu et al., 2009). Induced a decreased expression of methyl-binding protein MeCP2 in prefrontal cortex and striatum (Kim et al., 2013)	DNA methylation variation in genes related to neurodevelopmental and peroxisomal processes (Walton et al., 2016); Cytosine methylation (Mill and Petronis, 2008); Several genes methylated: DAT1 (Ding et al., 2016); IGF2 (Rijlaarsdam et al., 2016); 5-HT3A R (Perroud et al., 2016); SLC6A4 (Park et al., 2015)
chromatin configuration	Down-regulation of 104 genes involved in protein synthesis, mRNA splicing, and chromatin organization (Rogic et al., 2016)	Histone modification in ADHD (Mill and Petronis, 2008); histone acetylation increased significantly in the hippocampus by chronic lead exposure, causing ADHD-like symptoms (Luo et al., 2014)
microRNA	PAE is associated with dysregulation of several miRNA levels (Balaraman et al., 2013; Gardiner et al., 2016)	Small interfering RNA (siRNA) is involved in ADHD (Mill and Petronis; (Kandemir et al., 2014)); abnormal miRNA function contributes to ADHD (Wu et al., 2015; Garcia-Martínez et al., 2016; Ye et al., 2016)

## Behavioral similarities and differences between ARND of FASD, derived from PAE and ADHD

The prognosis and treatment responses for children with ADHD and FASD differ to those of children with ADHD alone. Individuals with FASD may respond differently to stimulant medication than other children with ADHD. For example, there is evidence suggesting that children with FASD and ADHD have a better response to dexamphetamine than to methylphenidate (O'Malley et al., 2000).

Executive function is a core deficit in FASD and ADHD, common symptoms of behavioral disinhibition and attention deficit may be related to problems with it. So, children with FASD and ADHD may have structural and functional abnormalities in the frontal-subcortical circuits, which are areas associated with executive function. Both populations have deficits in global adaptive abilities and adaptive behavior is affected in both FASD and ADHD (Crocker et al., 2009), however, the children with PAE performed worse than the ADHD group on letter and category fluency (Vaurio et al., 2008).

Children with PAE had similar global intellectual deficits to children with ADHD. Nevertheless, the PAE group struggled on arithmetic, while ADHD group were poorer on reading/decoding. Additionally, by using a four-factor model that specifically analyzes attention through a group of four processes and links them to a putative system of cerebral structures (Mirsky et al., 1991; Kremen et al., 1992), the PAE group encountered problems with encoding and shift, while the ADHD group had difficulties with focus and sustain (Coles et al., 1997); thus, their neurocognitive deficits may not be completely the same.

Factor analyses of ADHD symptoms divide its behavioral symptoms into two separate domains, one reflecting inattention and the other, a combination of hyperactivity and impulsivity (Toplak et al., 2012). To date, the best-validated animal models for ADHD are the Spontaneously Hypertensive Rat (SHR/NCrl) (Rat Genome Database, 2016)^2^ and the Wistar KYoto rat (WKY/NCrl) (Charles River, Germany) for the inattentive phenotype (Sagvolden and Johansen, 2012; Zhang-James et al., 2013). According to Sagvolden (2000) and Sagvolden et al. (2009), because the diagnosis of ADHD is based on behavior, validation of animal models must also be based on behavior. Therefore, if valid animal models were to be found, one would expect many of the same fundamental genetic and neurobiological alterations to be common in the case of humans and animals.

In rodents, PAE can produce an increase of locomotor activity and attentional demand, as well as an attentional deficit, analogous to those observed in FAS and ADHD (Brys et al., 2014). Additionally, PAE induced hyperactive, inattentive, and impulsive behavioral phenotypes in mouse and rat offspring, with increased expression of DAT in prefrontal cortex and striatum (Kim et al., 2013), which constitute an additional possible link between FASD and ADHD-like behavioral phenotypes.

There is limited information on the genetic influences of inattention. Transcriptional profiling analysis in animal models of disorders may provide an important tool to identify genetic involvement in behavioral phenotypes (De la Peña et al., 2015). Consequently, there is a need for large, high quality studies examining the etiology, diagnosis, and interventions for ADHD within FASD. By improving the understanding of the etiology of ADHD within FASD, will develop more effective interventions and the ability to diagnose FASD more accurately (Peadon and Elliott, 2010).

## Concluding remarks and perspectives

Prenatal Alcohol Exposure (PAE) is a major, preventable cause, of induced CNS defects during development named as “Alcohol Related Neurodevelopmental Disorders” (ARND), that undoubtedly gives rise to behavioral and cognitive deficits in children. It also affects child's growth and even more, it is able to induce morphological alterations (facial features), generating a broader “Fetal Alcohol Spectrum Disorders” (FASD). On the other hand, ADHD is a chronic condition that affects a relevant percentage of children (between 5 and 7%) and that often continues into adulthood. Spite some specific and punctual characteristic differences between them, the prevalence of ADHD (diagnosed according to DSM-IV criteria) in children with heavy PAE is extremely high (Bhatara et al., 2006; Fryer et al., 2007). Therefore, a possible causal effect relationship, or at least a physiological parallelism can be deducted between ARND and ADHD (summarized in Table 1).

Attention-deficit/hyperactivity disorder (ADHD) includes a combination of persistent problems, such as difficulty sustaining attention, hyperactivity and impulsive behavior. Being also these aspects alterated in ARND after PAE. Consequently, it is reasonable the idea that animal models that exhibits these characteristics and additionally affects similar CNS circuits and neuronal systems, as PAE, can be suitable models for the analysis of the molecular and behavioral basis of those alterations. Finally, it is fundamental to clearly establish several aspects of the specific protocols applied, both for PAE and for the behavioral analysis of their consequences, as well as for ADHD characterization.

Then, although there is abundant literature regarding the study of PAE and ADHD cognitive and behavioral effects, nonetheless, the exact etiological factors as well as the underlying molecular mechanism of pathophysiogy affected by them has not been fully understood yet. Given the complexity of the effects elicited by PAE, it is mandatory to perform a more integral experimental approach, as well as, a wider open gene expression analysis, including epigenetic alterations, in order to identify the molecular mechanisms, parenthetically the molecules, involved in each step of the affection, and to provide a clear cellular and molecular substrate that further explains the cognitive and behavioral alterations. In this regard, increasing the knowledge of the molecular basis involved, would lead us to a clearer identification of the subjacent cellular and molecular mechanisms, and therefore serve as a guide to improve not only the behavioral characterization, but also to a higher progress in the diagnoses and treatment for those pathological conditions. Then, experimental paradigms in animal models focusing on the study of the PAE effects at cellular and molecular levels during embryonic (fetal) development, in correlation with postnatal behavioral and cognitive test, in young and adults, are warranted, and would elicit relevant information about ADHD condition.

## Author contributions

AR conceived the work. AR and EP acquire and compiles information. AR and DO drafted and revised critically. All authors reviewed the paper and approved the final version.

### Conflict of interest statement

The authors declare that the research was conducted in the absence of any commercial or financial relationships that could be construed as a potential conflict of interest.

## Footnotes

^1^NIH definition: http://www.nimh.nih.gov/health/topics/attention-deficit-hyperactivity-disorder-adhd/index.shtml.

^2^Rat Genome Database (2016). http://rgd.mcw.edu.
